# Performance evaluation of a new fourth-generation HIV combination antigen–antibody assay

**DOI:** 10.1007/s00430-012-0250-5

**Published:** 2012-06-17

**Authors:** A. Mühlbacher, H. Schennach, J. van Helden, T. Hebell, G. Pantaleo, P. Bürgisser, C. Cellerai, P. Permpikul, M. I. Rodriguez, A. Eiras, F. Alborino, P. Cunningham, M. Axelsson, S. Andersson, O. Wetlitzky, C. Kaiser, P. Möller, G. de Sousa

**Affiliations:** 1 Central Institute for Blood Transfusion and Immunology, Innsbruck, Austria; 2 MVZ Stein und Partner, Mönchengladbach, Germany; 3 MVZ Wagnerstibbe für Laboratoriumsmedizin, Gynäkologie, Humanmedizin und Pathologie GmbH, Göttingen, Germany; 4 Département de Pathologie et de Médecine de Laboratoire, Service D’Immunologie et Allergie, Centre Hospitalier Universitaire Vaudois, Lausanne, Switzerland; 5 Department of Transfusion Medicine, Faculty of Medicine Siriraj Hospital, Mahidol University, Bangkok, Thailand; 6 Centro de Transfusion de Galicia, Santiago de Compostela, Spain; 7 Ospedaliero di Dolo, Servizio di Medicina di Laboratorio, Dolo, Italy; 8 St. Vincent’s Centre for Applied Medical Research, St. Vincent’s Hospital Sydney, Darlinghurst, NSW Australia; 9 Department of Virology, Swedish Institute for Infectious Control SMI, Solna, Sweden; 10 MVZ Labor München Zentrum, Munich, Germany; 11 Hospital Fernando Da Fonseca, Lisbon, Portugal

**Keywords:** HIV, Immunoassay, Qualitative, Diagnostic

## Abstract

Education and diagnostic tests capable of early detection represent our most effective means of preventing transmission of human immunodeficiency virus (HIV). The importance of early detection is underlined by studies demonstrating increased life expectancy following early initiation of antiviral treatment. The Elecsys^®^ HIV combi PT assay is a fourth-generation antigen–antibody combination assay developed to allow earlier detection of seroconversion, and to have increased sensitivity and improved specificity. We aimed to determine how early the assay could detect infection compared with existing assays; whether all HIV variants could be detected; and the assay’s specificity using samples from blood donors, routine specimens, and patients with potential cross-reacting factors. Samples were identified as positive by the Elecsys^®^ assay 4.9 days after a positive polymerase chain reaction result (as determined by the panel supplier), which was earlier than the 5.3–7.1 days observed with comparators. The analytical sensitivity of the Elecsys^®^ HIV combi PT assay for the HIV-1 p24 antigen was 1.05 IU/mL, which compares favorably with the comparator assays. In addition, the Elecsys^®^ assay identified all screened HIV subtypes and displayed greater sensitivity to HIV-2 homologous antigen and antibodies to HIV-1 E and O and HIV-2 than the other assays. Overall, the specificity of the Elecsys^®^ assay was 99.88 % using samples from blood donors and 99.81 % when analyzing unselected samples. Potential cross-reacting factors did not interfere with assay performance. The Elecsys^®^ HIV combi PT assay is a sensitive and specific assay that has been granted the CE mark according to Directive 2009/886/EC.

## Introduction

Human immunodeficiency virus (HIV) is a member of the retrovirus family. The virus is found worldwide but is most common in Africa, with a prevalence of >15 %; prevalences of 0.5 to <1.0 % and 0.1 to <0.5 % have been reported for the USA and UK, respectively [1]. The virus evolves rapidly, and two main types have been identified: HIV-1 and HIV-2, with HIV-1 being responsible for infecting the majority of patients. HIV-1 is further subdivided, based on genetic variation, into three main groups, M (major), O (outlier), and N (non-M, non-O), with type M having nine subtypes (A, B, C, D, F, G, H, J, and K); there are then at least six sub-subtypes (A1–A4, F1, and F2) [2, 3]. These HIV groups and subtypes have a distinct geographic distribution. HIV-2 is less infective and is generally confined to particular parts of, or areas with a strong association with, West Africa; in North America and Western Europe, HIV-1 group M subtype B is the most prevalent subtype, while subtype A is most common in Eastern Europe and Central Asia, and subtype C is most common in India. In addition, some variants result from a recombination event between two or more different subtypes, and a new group of HIV-1, group P, has recently been identified [2, 4].

Transmission of HIV is by contact with bodily fluids, such as blood, semen, and vaginal secretions, and can occur via unprotected sex, unscreened blood transfusions, or through sharing needles [3]. HIV can also be transmitted from mother to child during pregnancy, delivery, or by breast-feeding [5]. Once infected, patients remain infected for life as the viral genome integrates into the DNA of host cells. The major target cells are CD4+ T lymphocytes that are a vital part of the immune system. The function of the immune system becomes compromised as these cells are destroyed [3].

During the first few weeks after infection, patients can experience non-specific influenza-like symptoms, such as fever and aching muscles, which resolve within a month. During this time, known as the primary infection (often 1–3 weeks, but sometimes up to 10 weeks after infection), there is a peak in viral RNA and p24 antigen that is detectable in the patient’s serum. Seroconversion occurs in general about 25 days after infection, and immunoglobulin (Ig) M followed by IgG antibodies to viral proteins become detectable in serum. A next phase follows in which the patient is asymptomatic while viral RNA in the plasma declines and stabilizes at a value called ‘viral set point’, and p24 antigen is no longer detectable. In the final phase, and once the immune system has been compromised, patients become susceptible to opportunistic infections and exhibit the symptoms known as the acquired immunodeficiency syndrome (AIDS). At this stage of the disease, the viral RNA level rises again, while p24 antigen may reappear and antibodies to viral *gag* and *pol* proteins may no longer be detectable in serum samples from the patient [6, 7].

Given the lack of a cure for HIV infection, preventing transmission is paramount. The highest risk of transmission is during the very early stage of infection due to the high concentration of HIV in the blood and genital secretions [8]. Furthermore, the patient is probably unaware that he/she is infected and so may not be taking precautions. Also, despite the lack of symptoms during the asymptomatic phase, medium to high concentrations of HIV are often present in the blood leading to a continued high risk of transmission. Hence, education regarding prevention of transmission and tests capable of detecting as soon as possible whether or not someone is infected are important factors in the management of HIV prevention.

Although there is no cure, viral suppression with antiviral therapy can maintain immune function and reduce both mortality and the effect of opportunistic infections [9]. Early initiation of treatment has been shown to increase life expectancy, adding to the need to detect infection early in the disease course [10].

The fourth-generation assays were developed to allow earlier detection of HIV seroconversion and reduce the time period to positive virus detection [11–13]. More recently, the Elecsys^®^ HIV combi PT assay (Roche Diagnostics, Penzberg, Germany) has been developed as an update to the previous assay; it differs from the Elecsys^®^ HIV combi assay as it includes a pre-treatment step to improve specificity and increase sensitivity to HIV-1 p24 antigen, thereby improving early detection of HIV infection. This assay contains a special set of anti-p24 antibodies that allow early detection of infection, late-phase infection, and detection of p24 antigen derived from HIV-1 group O and HIV-2. In addition, the assay contains a set of antigens including gp41, gp36, HIV-1 RT, and HIV-2 RT in order to provide high sensitivity to anti-HIV-1 and anti-HIV-2 antibodies, as well as enhanced security for detecting antibodies against all subtypes (including circulating recombinant forms of HIV and HIV-1 subtype O). Other fourth-generation HIV assays are also available, such as the ARCHITECT^®^ HIV Ag/Ab combo (Abbott Laboratories, Wiesbaden, Germany), AxSYM^®^ HIV Ag/Ab combo (Abbott Laboratories, Wiesbaden, Germany), and ADVIA Centaur^®^ HIV Ag/Ab combo (Siemens Healthcare Diagnostics Inc, Deerfield, USA) assays.

The aim of this study was to determine whether the Elecsys^®^ HIV combi PT assay can reliably detect infection with all investigated HIV variants, and at the earliest possible stage of infection. The specificity of the assay using samples from blood donors, routine specimens, and patients with potential cross-reacting factors (such as from patients with elevated rheumatoid factor (RF), autoantibodies or monoclonal gammopathy, or other viral infections) was also determined.

## Materials and methods

The study was carried out at 12 centers: Central Institute for Blood Transfusion and Immunology, University Hospital Innsbruck, Innsbruck, Austria; MVZ Stein und Partner, Mönchengladbach, Germany; MVZ Wagnerstibbe für Laboratoriumsmedizin, Gynäkologie, Humanmedizin und Pathologie GmbH, Göttingen, Germany; Département de Médecine de Laboratoire, Service d’Immunologie et Allergie, Centre Hospitalier Universitaire Vaudois, Lausanne, Switzerland; Department of Transfusion Medicine, Siriraj Hospital and Medical School, Mahidol University, Bangkok, Thailand; Centro de Transfusion de Galicia, Santiago de Compostela, Spain; Ospedaliero di Dolo, Servizio di Medicina di Laboratorio, Dolo, Italy; St. Vincent’s Centre for Applied Medical Research, St. Vincent’s Hospital Sydney, Darlinghurst NSW, Australia; Swedish Institute for Infectious Control SMI, Department of Virology, Solna, Sweden; MVZ Labor München Zentrum, Munich, Germany; Hospital Fernando Da Fonseca, Lisbon, Portugal; and Roche R&D, Penzberg, Germany.

### Elecsys^®^ HIV combi PT assay

The assay is performed according to the sandwich principle. During the first incubation, any HIV particle available within the sample is lysed to release the p24 antigen. During the second incubation, a sandwich complex is formed between anti-HIV antibodies and the biotin- and ruthenium-labeled HIV-specific antigens, and between p24 antigen and the biotin- and ruthenium-labeled monoclonal anti-p24 antibodies. Following the addition of streptavidin-coated microparticles, the third incubation allows the complexes to bind to the solid phase as a result of the interaction between biotin and streptavidin. The bound microparticles are then magnetically captured onto the surface of an electrode, and unbound material is removed. Chemiluminescent emission is induced by applying a voltage to the electrode and measured by a photomultiplier. The results are automatically determined by the Elecsys^®^ software that compares the signal produced from the sample with the cutoff value obtained during system calibration. The total assay time is 27 min [14].

In this study, the Elecsys^®^ HIV combi PT assay was performed on the Elecsys^®^ 2010, e411, or MODULAR *Analytics* ^®^ E170 immunoassay analyzer.

### Comparator assays

The comparator assays used, namely the Abbott ARCHITECT^®^ HIV Ag/Ab combo, Abbott AxSYM^®^ HIV Ag/Ab combo, Abbott PRISM^®^ HIV O Plus, and Siemens ADVIA Centaur^®^ HIV Ag/Ab combo assays, are registered with European Notified Bodies and represent state-of-the-art HIV screening assays. Assays were performed and interpreted according to the manufacturers’ instructions. Each assay is described briefly below.

The ARCHITECT^®^ HIV Ag/Ab combo assay uses monoclonal mouse antibodies and HIV antigen-coated microparticles to bind the antigens and antibodies within the sample. These are then bound to acridinium-labeled conjugates, and a trigger solution is added to initiate a chemiluminescent reaction that is measured by the ARCHITECT^®^ system [15].

The AxSYM^®^ HIV Ag/Ab combo assay also uses microparticle enzyme immunoassay technology. Recombinant *E. coli* HIV antigens and mouse anti-HIV p24 monoclonal antibodies coated on microparticles bind HIV antigens and antibodies from the sample. Biotin-labeled recombinant antigens, peptides, and anti-p24 monoclonal antibodies react with the captured antibodies/antigens and are detected using an anti-biotin:alkaline phosphatase conjugate; the fluorescence produced on the addition of 4-methylumbelliferyl phosphate is measured by the AxSYM^®^ system [16].

The PRISM^®^ HIV O Plus assay detects HIV antibodies using a three-step sandwich procedure. Microparticles coated with HIV antigens are used to bind to antibodies in the sample and are captured on a glass fiber matrix. Biotinylated peptides and proteins and an acridinium-labeled anti-biotin conjugate are added, followed by an alkaline hydrogen peroxide solution to generate a chemiluminescent signal [17].

In the ADVIA Centaur^®^ HIV Ag/Ab combo assay, antibodies or antigens within the patient sample are bridged to antigens and antibodies provided with the assay. Streptavidin-coated microparticles and biotinylated recombinant antigens are used to capture the antigens and antibodies, and acridinium ester-labeled recombinant antibodies/antigens are then added to generate relative light units for detection on the ADVIA Centaur^®^ analyzer [18].

### Samples

Samples stored in aliquots at −20 °C were used for sensitivity testing, and only one freeze/thaw cycle was permitted. Fresh samples from blood donors and samples taken routinely from hospitalized patients and patients with potential cross-reacting factors were tested in parallel with the comparator assay. Sensitivity and specificity were determined using the following sample types.

### Commercial seroconversion panels

Seroconversion panels used to analyze early infection were purchased from SeraCare Life Sciences Inc (Milford, MA, USA; *n* = 8: PRB929, PRB930, PRB940, PRB943, PRB957, PRB964, PRB965, PRB966) and Zeptrometrix Inc (Buffalo, NY, USA; *n* = 6: HIV 9012, HIV 9016, HIV 9021, HIV 9023, HIV 9034, HIV 6243). These panels comprised samples taken from patients before and after they tested positive for HIV infection and throughout the immune response.

### Samples from patients in the early stage of infection

Sequential samples taken from patients soon after infection were available to the centers in Lausanne (59 samples from 24 patients) and Sydney (30 samples from 15 patients) and were used to assess sensitivity.

### NIBSC/WHO p24 antigen standard

The Elecsys^®^ HIV combi PT, ARCHITECT^®^ HIV Ag/Ab combo, ADVIA Centaur^®^ HIV Ag/Ab combo, and AxSYM^®^ HIV Ag/Ab combo assays were assessed against the NIBSC/WHO p24 antigen standard (NIBSC 90/636) using the following dilutions: 0.000, 0.625, 1.250, 2.500, 5.000, and 10.000 IU/mL. Samples were diluted with human donor plasma that was negative for all HIV serologic markers.

### HIV subtypes

Samples were known to be antibody positive for HIV-1 group M subtypes (*n* = 609), HIV-1 group O subtypes (*n* = 8), or HIV-2 (*n* = 472) as a result of immunoblot testing, sequencing the *pol* amplicon spanning the whole protease and first 1,200 nucleotides of the reverse transcriptase open reading frame, or using an immunological in vitro enzyme test for determining HIV-1 subtypes A–E.

In addition, serial dilutions of 28 lysate samples derived from culture supernatants and representing different HIV variants were tested. The lysates were known to contain antigens from HIV-1 subtypes A–H, O, or HIV-2, or antibodies to HIV-1 subtypes A–G, O, or HIV-2.

### Blood donations

Fresh serum samples or EDTA plasma from unselected blood donors were used for the specificity analyses. Any discrepant sample was retested, and a confirmatory assay was used for any repeatedly reactive sample.

### Daily routine samples

Fresh serum or EDTA plasma samples collected from hospitalized patients, outpatients, pre-surgery patients, dialysis patients, pregnant women, and for anonymous testing were used for the specificity analyses. All samples were unselected.

### Potential cross-reaction

A total of 148 samples from patients containing potential cross-reacting factors were used. These samples were selected as they were from patients with RF >200 IU/mL, autoantibodies or monoclonal gammopathy, or who had antibodies to other viral infections that may have interfered with the performance of the assays (Epstein–Barr virus IgM, Epstein–Barr virus IgG, cytomegalovirus IgM, cytomegalovirus IgG, herpes simplex virus IgM, herpes simplex virus IgG, and influenza vaccine).

### Data analysis

Prior to the start of all evaluations, each center performed a system and reagent familiarization with run-to-run, inter-assay precision.

Results were obtained from single measurements where possible. Initially reactive samples were retested in single measurements or double determinations, while repeatedly discrepant and positive samples were measured against confirmatory assays. Results were expressed as s/co ratios. Results were deemed to be negative if the s/co <0.9 and positive if the s/co ≥1.0. An s/co between 0.9 and <1.0 was considered to fall within a ‘gray zone’; this 10 % gray zone was introduced during the data validation, and samples in this zone were considered positive. Results obtained from the comparator assays were interpreted according to the information provided by the manufacturer [15–18].

Data on early detection were calculated using the Paul Ehrlich Institute model, as reported by Couroucé et al. [19]. Seroconversion panels comprising serial samples from patients before and after they tested positive for HIV infection were assessed using the immunoassays. The PCR data used to determine positivity were provided by the panel suppliers and were obtained using the following assays: Procleix^®^ HIV-1/HCV assay (Gen-Probe Inc.), COBAS^®^ Amplicor HIV-1 Monitor (Roche), HIV-1 bDNA (Chiron/Siemens), and COBAS^®^ AmpliPrep/COBAS^®^ TaqMan^®^ HIV Test v1.0 (Roche); a PCR test is the most sensitive for HIV detection and can detect HIV infection before seroconversion. The Paul Ehrlich Institute model calculates the time difference between the PCR test identifying the sample as positive and the immunoassay reporting a positive result. In this model, seroconversion is theoretically possible the day after the last negative test result [19]. Hence, if the PCR test and immunoassay both detect the same panel sample as positive, the difference is 0, whereas if the first sample identified by the immunoassay as positive was taken 4 days after the sample identified as positive by the PCR test, the difference is 4. The total and the average numbers of days of detection after a positive PCR result were calculated.

## Results

### Sensitivity: commercial seroconversion panels

A total of 14 seroconversion panels (comprising 135 samples) were analyzed to determine how soon after a positive PCR result the immunoassays could detect HIV infection. The results are given in Table 1 and show that the Elecsys^®^ assay detects HIV infection at a similar or shorter time interval than the comparator assays.

**Table 1:** Seroconversion sensitivity data using the Paul Ehrlich Institute calculation method

Seroconversion panel	Elecsys^®^ HIV combi PT	ADVIA Centaur^®^ HIV Ag/Ab combo	AxSYM^®^ HIV Ag/Ab combo	ARCHITECT^®^ HIV Ag/Ab combo
PRB929	0	4	0	0
PRB930	0	3	0	0
PRB940	0	7	0	0
PRB943	2	7	2	2
PRB957	2	9	14	9
PRB964	7	8	7	7
PRB965	7	7	12	5
PRB966	9	13	9	9
HIV 9012	16	16	16	16
HIV 9016	3	3	3	3
HIV 9021	4	4	4	4
HIV 9023	7	7	7	7
HIV 9034	5	5	5	5
HIV 6243	7	7	7	7
Total number of days	69	100	86	74
Mean number of days	4.929	7.143	6.143	5.286

Day of bleeding with first positive results (last negative sample + 1 day) compared with PCR data provided by the panel suppliers (SeraCare and Zeptometrix). The first positive bleed with the PCR assay was considered to be day 0

Additional data calculated using all assessments of the Elecsys^®^ HIV combi PT assay are available for a total of 90 seroconversion panels; these results were calculated irrespective of the comparator assay used (and, in some cases, a comparator was not used). These data support the findings given in Table 1 and show that the total number of days to a positive Elecsys^®^ test result after a positive polymerase chain reaction (PCR) result was 493 days, giving a mean delay of 5.478 days (Roche, data on file).

### Sensitivity: samples from patients in the early stage of infection

Samples from 15 patients were tested using the Elecsys^®^ HIV combi PT and ARCHITECT^®^ HIV Ag/Ab combo assays by the group in Sydney. Both assays identified 29 HIV-positive bleedings out of a total of 30 bleedings, giving a number of patient panels/positive bleedings of 0.517.

The group in Lausanne tested 59 samples from 24 patients using the Elecsys^®^ HIV combi PT assay and identified 58 as HIV positive giving a number of patient panels/positive bleedings of 0.414. Of these 24 patients, 19 had detectable HIV p24 antigen but were negative for IgM and IgG (pre-seroconversion), and two were positive for IgM before being positive for IgG (IgM seroconversion); for two patients, it was not possible to distinguish between the previous two categories. The remaining patient was at a later stage of seroconversion. Samples from one patient were found to be positive on day 0 but negative on day 13.

### Sensitivity: NIBSC/WHO p24 antigen standard

Results using the NIBSC/WHO p24 antigen standard dilution series and the Elecsys^®^ HIV combi PT assay showed a linear relationship. The analytical sensitivity of the Elecsys^®^ HIV combi PT assay was calculated to be 1.05 IU/mL, which compares favorably with the comparator assays. The analytical sensitivities calculated during this study for comparator assays were the following: 0.94 IU/mL for the ARCHITECT^®^ HIV Ag/Ab combo; 1.89 IU/mL for the ADVIA Centaur^®^ HIV Ag/Ab combo; and 1.20 IU/mL for the AxSYM^®^ HIV Ag/Ab combo.

### Sensitivity: HIV subtypes

A summary of the sensitivity of the Elecsys^®^ HIV combi PT assay for detecting HIV subtypes is shown in Table 2. The assay correctly identified all investigated HIV-1 and HIV-2 subtypes.

**Table 2:** Summary of the sensitivity of the Elecsys^®^ assay for identification of HIV subtypes in antibody-positive samples

	Elecsys^®^ HIV combi PT (tested/positive)
HIV-1 group M subtypes	
A, A1, A2, A/B, A/C, A/E/C, CRF02_AG	72/72
B, B/A, B/C, CRF14_BG	262/262
C, C/A, C/B, C/E	168/168
D	16/16
E, E/A/C, E/C, E/D, CRF01_AE (=E)	59/59
F	7/7
F1	2/2
G	16/16
H	1/1
J	1/1
CRF06_cpx	1/1
CRF11_cpx	1/1
CRF13_cpx	3/3
HIV-1 group O subtypes	8/8
HIV-2	472/472

The Elecsys^®^ assay recognized both antigen and antibodies to all investigated HIV subtypes over a series of dilutions (Table 3). In addition, the Elecsys^®^ HIV combi PT assay showed greater sensitivity than at least one of the comparator assays for detecting HIV-2 antigen and antibodies to HIV-1 E, O, and HIV-2.

**Table 3:** Dilution of lysate samples derived from culture supernatants at which the assays positively detect (s/co ≥1.0) specific HIV subtype antigen or antibody

HIV subtype	Elecsys^®^ HIV combi PT	ADVIA centaur^®^ HIV Ag/Ab combo	AxSYM^®^ HIV Ag/Ab combo	ARCHITECT^®^ HIV Ag/Ab combo
HIV antigen				
A	1:64,000	1:64,000	1:64,000	1:128,000
B	1:37,280	1:18,640	1:37,280	1:37,280
C	1:26,720	1:26,720	1:26,720	1:53,440
D	1:21,280	1:21,280	1:21,280	1:42,560
E	1:31,968	1:7,992	1:31,968	1:63,936
F	1:15,936	1:15,936	1:7,968	1:15,936
G	1:52,896	1:26,448	1:52,896	1:105,792
H	1:11,376	1:11,376	1:11,376	1:22,752
O	1:256	Failed to detect diluted antigen	1:128	1:512
HIV-2	1:64	Failed to detect diluted antigen	1:8	Failed to detect diluted antigen
HIV antibody^a^				
A	1:170,000	1:17,000	1:17,000	1:17,000
A	1:5,000	1:5,000	1:5,000	1:500
B	1:15,000	1:1,500	1:1,500	1:1,500
B	1:120,000	1:120,000	1:120,000	1:120,000
C	1:100,000	1:10,000	1:100,000	1:10,000
C	1:200,000	1:200,000	1:200,000	1:200,000
D	1:50,000	1:5,000	1:5,000	1:5,000
D	1:28,500	1:28,500	1:28,500	1:2,850
E	1:50,000	1:500	1:5,000	1:500
E	1:50,000	1:5,000	1:5,000	1:500
F	1:22,800	1:228	1:2,280	1:2,280
F	1:100,000	1:100,000	1:100,000	1:100,000
G	1:35,000	1:35,000	1:35,000	1:3,500
G	1:140,000	1:140,000	1:14,000	1:14,000
O	1:10,000	Failed to detect diluted antibody	1:10,000	1:1,000
O	1:500,000	Failed to detect diluted antibody	1:5,000	1:5,000
HIV-2	1:50,000	1:500	1:5,000	1:500
HIV-2	1:60,000	1:600	1:6,000	1:6,000

^a^Two different antibody samples were tested for each subtype

### Specificity: samples from blood donors

The specificity of the Elecsys^®^ HIV combi PT assay determined using samples from blood donors at the individual centers was as follows: Innsbruck, 99.86 % (*n* = 2,834); Bangkok, 99.93 % (*n* = 1,500); Santiago de Compostela, 99.87 % (*n* = 1,524); and Dolo, 99.87 % (*n* = 1,485).

Overall, the specificity of the Elecsys^®^ HIV combi PT assay was 99.88 % (*n* = 7,343) and showed a clear discrimination between positive and negative samples. In addition, the specificity of the Elecsys^®^ HIV combi PT assay using samples from blood donors was comparable to that of other assays; examples of the comparisons are shown in Table 4.

**Table 4:** Specificity analyses of samples from blood donors; data from Bangkok, Innsbruck, and Santiago de Compostela

	Bangkok		Innsbruck		Santiago de Compostela	
	Elecsys^®^ HIV combi PT	ARCHITECT^®^ HIV Ag/Ab combo	Elecsys^®^ HIV combi PT	ARCHITECT^®^ HIV Ag/Ab combo	Elecsys^®^ HIV combi PT	PRISM^®^ HIV O Plus^a^
Total number	1,500	1,500	2,834	2,834	1,524	1,524
RR ≥1 s/co	3	3	4	1	2	1
RR ≥0.9 s/co to <1.0 s/co	3	N/A	0	N/A	1	N/A
Immunoblot confirmed positive/PCR positive	2^b^	2^b^	0	0	0	0
Number of false positives	1	1	4	1	2	1
Specificity % RR ≥1 s/co	99.93	99.93	99.86	99.96	99.87	99.93
Lower confidence limit (95 %; 2-sided) RR ≥1	99.63–100.00	99.63–100.00	99.64–99.96	99.80–100.00	99.53–99.98	99.63–100

*RR* repeatedly reactive, *N/A* not applicable

^a^The PRISM^®^ HIV O plus assay only detects HIV antibodies (not antigen)

^b^One sample was HIV PCR positive with a viral load of 699 copies/mL; results for this sample were s/co 38–39 using the Elecsys^®^ assay and s/co 1.6–1.9 with the ARCHITECT^®^ assay

### Specificity: routine samples

The specificity of the Elecsys^®^ HIV combi PT assay determined in routine samples at the individual centers was as follows: Mönchengladbach, 99.80 % (*n* = 1,000); Göttingen, 100 % (*n* = 1,004); Lausanne, 99.71 % (*n* = 347); Sydney, 100 % (*n* = 249); Munich, 99.42 % (*n* = 700); Lisbon, 99.78 % (*n* = 467); and Penzberg, 100 % (*n* = 336).

A total of 4,103 samples from unspecified patients, dialysis patients, and pregnant women were used to investigate the specificity of the Elecsys^®^ HIV combi PT assay. In this group, the overall specificity of the Elecsys^®^ HIV combi PT assay was found to be 99.81 % (repeatedly reactive). Examples of the specificity compared with other assays are shown in Table 5.

**Table 5:** Specificity analyses of unselected samples

	Lisbon		Mönchengladbach		Munich		Sydney		Göttingen	
	Elecsys^®^ HIV combi PT	ADVIA Centaur^®^ HIV Ag/Ab combo	Elecsys^®^ HIV combi PT	ADVIA Centaur^®^ HIV Ag/Ab combo	Elecsys^®^ HIV combi PT	AxSYM^®^ HIV Ag/Ab combo	Elecsys^®^ HIV combi PT	ARCHITECT^®^ HIV Ag/Ab combo	Elecsys^®^ HIV combi PT	ARCHITECT^®^ HIV Ag/Ab combo
Total number	467	467	1,000	1,000	700	700	249	249	1,004	1,004
RR ≥1 s/co	16	17	2	7	12	10	0	0	1	4
RR ≥0.9 s/co to <1.0 s/co	0	N/A	0	N/A	0	N/A	0	N/A	0	N/A
Immunoblot and/or HIV antigen confirmed positive	15	15	0	0	8	8	0	0	1	1
Number of false positives	1	2	2	7	4	2	0	0	0	3
Specificity % RR ≥1 s/co	99.78	99.56	99.80	99.30	99.42	99.71	100.00	100.00	100.00	99.70
Lower confidence limit (95 %; 2-sided) RR ≥1	98.77–99.99	98.41–99.95	99.28–99.98	98.56–99.72	98.53–99.84	98.96–99.96	98.53–100.00	98.53–100.00	99.63–100.00	99.13–99.94

Results from five centers participating in the study (Lisbon, Mönchengladbach, Munich, Sydney, and Göttingen)

*RR* repeatedly reactive, *N/A* not applicable

### Specificity: potential cross-reaction

Samples from patients with potential cross-reacting factors were tested to analyze specificity. The Elecsys^®^ HIV combi PT assay correctly identified the majority of the samples as being HIV negative (147/148). The samples tested were from patients with RF elevation >200 IU/mL (21/21 HIV negative), autoantibodies (various; 21/21 HIV negative) or monoclonal gammopathy (20/21 HIV negative), or from patients positive for the following (but HIV negative): Epstein–Barr virus IgM (10/10 HIV negative), Epstein–Barr virus IgG (10/10 HIV negative), cytomegalovirus IgM (10/10 HIV negative), cytomegalovirus IgG (15/15 HIV negative), herpes simplex virus IgM (10/10 HIV negative), herpes simplex virus IgG (15/15 HIV negative), and influenza vaccine (15/15 HIV negative).

## Discussion

In this study, the Elecsys^®^ HIV combi PT assay showed good sensitivity and specificity in a wide range of samples and compared well with existing assays.

Early detection of infection is vital for patient management and the prevention of HIV transmission. The fourth-generation assays can detect HIV nearly 6 days before the third-generation assays and sometimes before p24 antigen-only assays, and less than 3 days after nucleic acid tests [20, 21]. In a previous report by Kwon et al., the ARCHITECT^®^ combo assay detected infection 4–26 days earlier than third-generation assays [22]. In our study, positive samples could be identified by the Elecsys^®^ HIV combi PT assay as early as 4.9 days after a positive PCR result (using the Paul Ehrlich Institute methods as described by Couroucé et al. [19]), compared with 5.3–7.1 days for the comparator assays (not significant).

The Elecsys^®^ HIV combi PT assay has been standardized to the WHO reference standard for p24 antigen and has an overall analytical sensitivity of 1.05 IU/mL at a cut-off index of 1.0. This is similar to, or lower than, some of the other assays; for example, in our study, the sensitivities of the Abbott ARCHITECT^®^ combo and AxSYM^®^ assay were 0.94 and 1.20 IU/mL, while a previous report gives sensitivities of 1.24 and ~2 IU/mL, respectively, for these two assays [23]. In addition, an analytical sensitivity of 1.05 IU/mL conforms to the stringent standard required in some countries (<2 IU/mL).

The Elecsys^®^ HIV combi PT assay identified all investigated HIV subtypes. Results from the lysate dilution series showed the assays to be comparable for detecting HIV-1 A–H and O capsid antigens but that the Elecsys^®^ HIV combi PT assay had greater sensitivity for detecting HIV-2 antigen. With regard to sensitivity for detecting antibody, the Elecsys^®^ HIV combi PT assay recognized antibodies to HIV-1 E, O, and HIV-2 with greater sensitivity than the other assays, and comparable sensitivity for antibodies to the remaining subtypes.

Previous studies investigating laboratory-based (as opposed to point-of-care based) HIV assays have documented that HIV-1 O strains and HIV-1 subtypes F and C have not been detected by some assays, and the difficulty in diagnosing HIV-1 group O has also been highlighted by the case described by Henquell et al. [20, 21, 24, 25]. In the report by Malm et al. [13], the Abbott AxSYM^®^ assay failed to detect one HIV-2 sample. In contrast, in our study the Elecsys^®^ HIV combi PT assay correctly identified as positive all samples containing HIV, irrespective of strain and subtype. Genetic variability, particularly of gp41, may affect the ability of enzyme assays to detect HIV antibodies [26]. For this reason, additional components were included in the Elecsys^®^ HIV combi PT assay in addition to a pre-treatment step; our results confirm that the modifications have enhanced the specificity and sensitivity of the assay.

In the context of blood donation screening, a highly sensitive algorithm for detecting HIV must be used to prevent HIV transmission via transfusions. It is possible that the Elecsys^®^ HIV combi PT assay could be considered for this purpose, given its high sensitivity and early detection rate. Furthermore, the assay has been shown to reliably detect all investigated HIV subtypes, including HIV-2, and has good specificity in the samples from blood donors tested to date.

Some studies have suggested that the fourth-generation assays are associated with the rare occurrence of a second diagnostic window during a period when antibody titers are low and there is a reduction in antigenemia. This phenomenon has been reported with several assays, including the AxSYM^®^ HIV Ag/Ab combo and Enzymun-Test^®^ HIV Combi assays [27–30]. One negative result during follow-up was obtained when testing the sensitivity of the Elecsys^®^ HIV combi PT assay using samples from patients in the early stage of infection. Overall, the Elecsys^®^ HIV combi PT assay detected more samples as being HIV positive earlier in the disease course, although a second diagnostic window can potentially occur with all fourth-generation assays. However, it is indeed a rare occurrence as more than 1,000 samples taken very soon after infection have been tested by the centers evaluating the assay and all HIV-positive samples were correctly identified.

Overall, the specificity of the Elecsys^®^ HIV combi PT assay using samples from blood donors was 99.88 %, with an overall specificity of 99.81 % when analyzing unselected samples. These results were comparable to those obtained with the other assays tested and with the previous reports. Malm et al. reported the specificities of the Abbott ARCHITECT^®^ and AxSYM^®^ assays to be 99.95 and 99.91 %, respectively, while other studies have suggested that fourth-generation assays have specificities ranging from 98 to 100 % [13, 20–22, 31, 32]. In addition, the Elecsys^®^ HIV combi PT assay demonstrated 100 % specificity in patients with infections other than HIV, such as cytomegalovirus or herpes simplex virus.

The Elecsys^®^ HIV combi PT assay is a sensitive and specific assay for the laboratory testing of HIV status, and a first positive result can be obtained on average 4.9 days after a PCR assay detects the presence of the virus in the context of primary infection. On the basis of the results presented here, the Elecsys^®^ HIV combi PT assay has been granted the CE mark according to Directive 2009/886/EC and is now available for use in laboratory-based testing [14].
